# A Whole Systems Approach to Hospital Waste Management in Rural Uganda

**DOI:** 10.3389/fpubh.2019.00136

**Published:** 2019-06-06

**Authors:** Stuart Kwikiriza, Alex G. Stewart, Birungi Mutahunga, Andrew E. Dobson, Ewan Wilkinson

**Affiliations:** ^1^Bwindi Community Hospital, Kanungu, Uganda; ^2^College of Life and Environmental Science, University of Exeter, Exeter, United Kingdom; ^3^Institute of Medicine, University of Chester, Chester, United Kingdom

**Keywords:** hazardous waste, compostable waste, SORT IT, operational research, mixed methods, personal protective equipment, zoonoses

## Abstract

**Introduction:** Safe waste management protects hospital staff, the public, and the local environment. The handling of hospital waste in Bwindi Community Hospital did not appear to conform to the hospital waste management plan, exhibiting poor waste segregation, transportation, storage, and disposal which could lead to environmental and occupational risks.

**Methods:** We undertook a mixed-methods study. We used semi-structured interviews to assess the awareness of clinical and non-clinical staff of waste types, risks, good practice, and concerns about hospital waste management. We quantified waste production by five departments for 1 month. We assessed the standard of practice in segregation, onsite transportation, use of personal protective equipment, onsite storage of solid waste, and disposal of compostable waste and chemicals.

**Results:** Clinical staff had good awareness of waste (types, risk) overall, but the knowledge of non-clinical staff was much poorer. There was a general lack of insight into correct personal or departmental practice, resulting in incorrect segregation of clinical and compostable waste at source (>93% of time), and incorrect onsite transportation (94% of time). In 1 month the five departments produced 5,398 kg of hazardous and non-hazardous waste (12; 88%, respectively). Good practice included the correct use of sharps and vial boxes and keeping the clinical area clear of litter (90% of the time); placentae buried immediately (>80% of the time); gloves were worn everyday by waste handlers, but correct heavy-duty gloves <33% of the time, reflecting the variable use of other personal protective equipment. Chemical waste drained to underground soakaways, but tracking further disposal was not possible. Correct segregation of clinical and compostable waste at source, and correct onsite transportation, only occurred 6% of the time.

**Conclusion:** Waste management was generally below the required WHO standards. This exposes people and the wider environment, including the nearby world heritage site, home to the endangered mountain gorilla, to unnecessary risks. It is likely that the same is true in similar situations elsewhere. Precautions, protection, and dynamic policy making should be prioritized in these hospital settings and developing countries.

## Introduction

Health care waste management is a global concern. All health care activities generate waste, which when poorly managed can affect the environment, the community, and domestic and wild animals. It is an issue of growing concern as the number of health care facilities is increasing while population growth reduces space for waste disposal (1). Waste generated by human activities and changes associated with lifestyles threatens both human beings and natural resources (1–3).

The World Health Organization (WHO) defines medical waste as waste generated by health care activities including a broad range of materials, from used needles and syringes to soiled dressings, body parts, diagnostic samples, blood, chemicals, pharmaceuticals, medical devices, and radioactive materials (4).

Health care waste is defined as all types of waste produced in health facilities such as hospitals, health centers, and pharmaceutical shops (2). The majority (85%) of the waste is non-hazardous, compostable/biodegradable, and non-compostable, which does not require specialist disposal. The remainder is hazardous waste: 10% infectious and highly infectious, and 5% is toxic chemicals, radioactive, and pharmaceuticals (5, 6), all of which requires special care and processing. Placentae are classed as highly infectious in settings such as Uganda, where blood-borne viruses are common, and need to be handled carefully (7).

Waste from health care activities can have a long-lasting impact on human health, including people handling the waste and the public in general (7–10) and the environment can be contaminated through underground water sources polluted by untreated medical waste buried in, or drained into, the ground (www.who.int/water_sanitation_health/medicalwaste/020to030.pdf).

People can be infected either through direct contact with contaminated waste or infected people, or indirectly via contamination of soil, ground water, surface water or air, or through affected animals. Direct or indirect exposure through environmental contamination by pharmaceutical and laboratory waste can also lead to disease, both in the human and animal populations (11–14).

Twenty-three percent of global deaths and 22% of global disability adjusted life years (DALYs) were attributable to environmental factors in 2012, including, but not limited to waste (15). Blood borne diseases like HIV and viral hepatitis B can be acquired through mismanagement of hazardous hospital waste.

In some industrialized countries, institutions that generate lots of waste, including health care waste, have a legal responsibility to manage such waste. As a result, they monitor the amount of hazardous waste generated and there are clearly organized structures for handling every type of waste. Different expensive and highly technical waste management methods are used, including solidification, elementary neutralization, carbon absorption, separation, filtration, and evaporation. This is as a result of considerable investment by authorities and organizations in waste handling and management, but these methods are not available in resource-poor countries. In these countries other, cheaper, but reasonably effective, methods like incineration, land filling, and composting are used to manage health care waste (16, 17).

## Background and Rationale

In low- and middle-income countries, health care waste management receives little attention as the health sector competes with other sectors of the economy for very limited resources. In most of these countries, health care waste is still handled and disposed of as domestic waste, with the resulting appreciable threat to the waste workers, the public, and the environment (5, 7, 18).

The literature about a whole systems approach to hospital waste management, from segregation of waste to disposal, that was relevant to rural, privately-funded hospitals in resource-poor countries, was limited (19). In a published paper from Uganda, waste generation rates in a public and a private hospital in Kampala, the capital city vary according to patients' circumstances (type and state of condition, number of people nursing a patient, number of visitors to a patient, items carried into ward) (8), but there is no clear mention of rural hospitals in a recent review across the developing world (1). The review concluded that the issue of health care waste management has received little attention and needs highlighting to create greater awareness.

In Uganda there is no legal framework requiring health facilities to take any special care with their waste disposal, and very limited finance available to address any such issues, either within the budgets of these facilities or from the government or other funding agencies. It is, therefore, possible that staff working in health facilities and people living nearby may be exposed to unnecessary risks, including possible environmental contamination (7, 15).

Bwindi Community Hospital, in southwest Uganda, has had its own waste management program since its inception in 2004 as part of its wide-ranging community health program. It generates heath care waste internally across departments, and externally during outreach health activities. The waste includes pathological, infectious, sharps, pharmaceutical, chemical, tissue, as well as non-infectious waste. The waste generated was thought to be systematically managed through a series of activities (including segregation at source, regular departmental collection, safe transport, storage, and disposal), to reduce the risk of any adverse outcome.

The hospital is located in a low land surrounded by forested hills of the impenetrable national park, a mile away, and several small water bodies, including one that generates hydro power that is supplied to the nearby trading center with a growing urban population in a radius of two kilometers. This is the first Uganda study in a rural hospital and such studies are still infrequent globally. Improper health care waste management can compromise health, safety and puts the environment at risk for all stakeholders in this community setting (1, 9).

This study evaluated the knowledge of clinical and non-clinical staff at Bwindi Community Hospital and assessed the current management of health care waste (hazardous waste—sharps, infectious, chemical, and pathological—and non-hazardous waste—compostable and non-compostable) during the month of October 2017.

Specifically, we (a) assessed the knowledge and practice of health care waste management by clinical staff and non-clinical staff, (b) measured the weight of waste generated and assess the effectiveness of the segregation of hazardous and non-hazardous waste in different clinical departments, (c) assessed the appropriate use of personal protective equipment by the porters, (d) reviewed the methods of on-site waste transportation, storage, and disposal of all waste, and (e) described the arrangements for offsite disposal of the hospital waste.

## Description of Case

Staff are trained when first employed to segregate waste at the point of generation by using color-coded bins with matched color-coded liners. Waste is collected daily from each department, except in two departments (Surgery and Sexual Reproductive Health) that produce a high volume of hazardous waste. In these two departments, waste is removed several times a day, after procedures have been carried out. Non-hazardous waste is separated into bins for compostable and non-compostable waste at the point of generation in the hospital.

Collection, including ensuring that all bin liners are securely closed, and transportation of waste to the storage site, is done by hospital porters, who should use appropriate personal protective equipment (gumboots, surgical face masks, heavy duty gloves, and plastic aprons).

The estates manager (SK) was aware of some shortcomings in the waste management system. Given the hospital vision, “*a healthy community free from preventable disease, and accessible health care for all,”* he realized that there could be wider implications in addition to the risk to hospital staff. It was therefore imperative to assess how the hospital waste was being managed and see if more could be done to ensure safe waste management, so as to mitigate the risks from pollution and infection.

## Methods

### Study Design

The study was a mixed-methods design, with a quantitative, descriptive, cross sectional study of waste management, with simultaneous qualitative in-depth interviews. This design was used to increase the breadth and depth of understanding of health care waste management.

### Setting

Uganda is a land locked country in East Africa, bordering the Democratic Republic of Congo, Rwanda, Tanzania, Kenya, and South Sudan. It has a population of 39 million people, half of whom are under 18 years. It is classed as a low-income country and 70% of the population are subsistence farmers (20). There are 165 hospitals in Uganda, with 40% government, 43% private not-for-profit, and 17% private for-profit (21).

Bwindi Community Hospital is a rural private not-for-profit hospital run by the Church of Uganda in Kanungu District, South-Western Uganda, with a large community health program, as reflected in the hospital vision. It is located over 500 kilometers from the capital city Kampala, and borders the Bwindi Impenetrable Forest National Park and the Democratic Republic of Congo. There is a poor road network, and no reliable source of power, or nearby facilities that can handle health care waste or recycling.

The community health program of the hospital includes health promotion, prevention, immunization, mental health, and support to over 500 community health volunteers. The volunteers are supported by 12 health centers and the hospital. The hospital provides general surgery, orthopedics, pediatrics, sexual, and reproductive health, adult inpatient and outpatient care.

### Study Population

The quantitative study population was hospital departments that generate waste. The qualitative study population was purposefully selected clinical and non-clinical staff directly involved in health care waste management.

### Data Variables and Sources

We assessed the knowledge and practice of health care waste management by clinical staff and non-clinical staff through semi-structured interviews. These in-depth interviews were conducted with purposive selection of staff (clinical and non-clinical) to elicit responses on the broad themes: segregation, collection and transport, disposal, risk, and concerns. Interviews were conducted by the principal investigator and another researcher, both experienced in qualitative methods, after obtaining written informed consent. An interview guide with open-ended questions was used. Interview questions focused on (a) types of waste generated (b) color coding for waste bins, (c) hazards posed by improper waste handling, (d) waste transportation, (e) storage and disposal, (f) concerns on waste handling (g) risk to population, and environment. All interviews were recorded with permission. Saturation was reached.

The quantitative data variables were each measured over 31 days, in October 2017, and included (a) the weight of each type of waste produced, and adequacy of its segregation by each clinical area, (b) if there was correct use of personal protective equipment by porters transporting the waste, (c) how waste bags were transported to the storage site, (d) if there was safe on-site storage and off-site removal of waste, (e) if the use of compost pits was appropriate, (f) if the disposal of laboratory and X-Ray chemicals was safe, and (g) if the burial of placentae and still-borne infants was appropriate and safe.

We also described the arrangements for offsite disposal of the hospital waste.

### Operational Definitions as Used in the Hospital

#### Correct Waste Management Practices

Acting according to hospital waste management guidelines: source segregation at generation points, proper transportation, and storage and disposal waste.

#### Segregation of Waste

The recognition and division of waste into the correct waste receptor.

#### Compostable Waste

Non-hazardous waste that will break down, safely, and relatively quickly, by biological decomposition.

#### Clinical Waste

Waste containing human tissue, blood, other body fluids, pharmaceutical products, or any items used directly in providing health services, unless rendered safe.

#### Hazardous Waste

Waste that poses any biological, chemical, radioactive or physical hazard.

#### Infectious Waste

Waste from patients with infections.

#### Highly Infectious Waste

Material used in patient care that is heavily contaminated by blood.

### Analysis

Interviews were recorded and transcribed and evaluated by two independent investigators to reduce bias and increase interpretive credibility. Any difference between the two was resolved by discussion to arrive at a consensus. A thematic network method, as described by Attride-Stirling, was used to analyze the data employing a global theme, organizing themes, and basic themes (20).

We undertook descriptive analysis of all quantitative data.

## Results

In total, over five tons (5,398 kg) of health care waste were produced by five departments of Bwindi Community Hospital in the study month. Of this, 12% (662 kg) was classed as hazardous and 88% (4,735 kg) as non-hazardous (Table 1).

**Table 1:** Type and weight of waste produced by clinical departments of Bwindi Community Hospital, Uganda, October 2017.

	**Sexual reproductive health department**		**Out patients department**		**Adult inpatient department**		**HIV/AIDS department**		**Pediatric ward**	
**Waste category**	**Mean daily waste in kg**		**Mean daily waste in kg**		**Mean daily waste in kg**		**Mean daily waste in kg**		**Mean daily waste in kg**	
**HAZARDOUS WASTE**										
-Highly infectious (Red color-code)	6.2		0.6		2.9		1.1		0.9	
-Infectious (Yellow color-code)	3.3		1.0		3.7		2.0		2.1	
**NON-HAZARDOUS WASTE**										
-Non-compostable waste	6.5		3.6		8.8		3.5		5.1	
-Compostable waste	45.1		11.1		34.3		6.7		31.7	
**Monthly total waste produced**	kg	%	kg	%	kg	%	kg	%	kg	%
Hazardous waste	299	16	46	10	127	9	98	24	92	8
Non-hazardous waste	1,597	84	429	90	1,297	91	30	76	1,107	92
All waste	1,896	100	475	100	1,425	100	404	100	1,199	100

The Sexual and Reproductive Health department produced over a third (35%) of the total waste in the study (Table 1). Adult Inpatients generated a quarter (26%) while Pediatrics produced a fifth (22%). HIV and Outpatients departments produced about 8% each.

Compostable waste, from food preparation by patients and their relatives, constituted nearly three quarters (3,902 kg, 72%) of the waste collected. This came particularly from the Sexual and Reproductive Health and Adult Inpatients.

Hazardous waste (highly infectious + infectious) made up 12% of all the waste in the study. The largest amount of hazardous waste was produced by the Sexual Reproductive Health and HIV departments.

**Figure 1:**
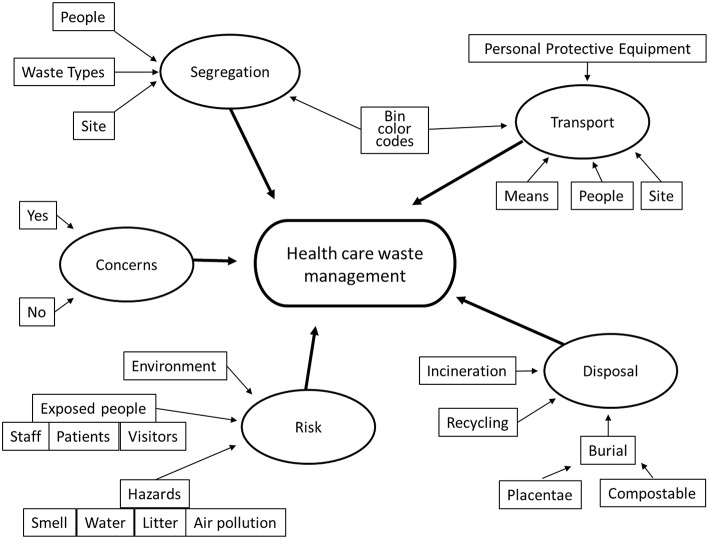
Thematic network showing the global theme (lozenge), organizing themes (ovals), and basic themes (rectangles) found through analyzing the qualitative interviews.

In-depth interviews were conducted with 15 clinical staff (nurses, midwives, clinical officers, lab staff, and medical doctors) and 6 non-clinical staff (administrators and porters). All interviewees had some knowledge about hospital waste types and gave examples. They knew the basics about hospital waste and the reasons why handling such waste is important. We report the findings under the organizing themes (segregation, transport, disposal, risk, and concerns) found through analysis of the interviews (Figure 1).

### Segregation

Waste should be collected in color-coded waste bins, with matching bin liners. There were sufficient waste-collecting bins throughout the hospital, but the correctly colored bin liners were often not available to order and so were not supplied consistently to the hospital wards. Staff used the available waste bins inconsistently. It was not clear if this was just them using the nearest available bin, expecting others to correctly segregate the waste later, or due to not being able to easily distinguish different bins (Table 2). A non-clinical staff member said, “*Training is one thing. Doing another.”* Segregation of waste was seen by some clinical staff as the job of the porters who transport the waste. “*If waste segregation is improved, the rest would be at rest*,” said a clinical staff member.

**Table 2:** Percentage of days with correct waste management practices by clinical departments in Bwindi Community Hospital, Uganda, October 2017.

	**Sexual reproductive health department**	**Out patients department**	**Adult inpatient department**	**HIV/AIDS department**	**Pediatric ward**
**Assessed waste management**	**%**	**%**	**%**	**%**	**%**
Segregation of clinical waste	7	0	3	3	4
Segregation of compost waste	3	3	7	7	7
Use of sharps & vial boxes	100	97	100	90	100
Clinical area clear of litter	100	100	100	97	97
Onsite-transportation of waste	3	3	3	7	3

*Number of days assessed varied between 29 and 31*.

However, the use of sharps and vial boxes and keeping clinical areas clean of litter showed good practice on most days (Table 2).

One clinical officer noted that there were no brown bin liners for pharmaceutical waste. This affects waste collection since pharmaceutical waste may be put in the wrong bins and may end up in the wrong disposal route. “*Supply [of brown bins] would put us at the level of people who handle waste very well*.

### Transport

Clinical waste (393 kg) was largely carried incorrectly by hand rather while non-clinical waste (3,903 kg) was transported within the hospital in a wheelbarrow. A clinical officer was concerned about the nature of waste transportation by porters: “*Waste is transported by porters on their backs*.” One non-clinical staff member said that he would like each department to have their own wheelbarrow for transporting waste because there is only one wheelbarrow in the hospital and most times it is in use, taking too long to become available.

Transportation to the storage facility was carried out incorrectly on most days (Table 2). The use of personal protective equipment by porters varied by equipment and between departments (porters are largely assigned to one department). While gloves were worn every day, the type of gloves worn were largely incorrect. Face masks were only used about a third of the time. The practice of wearing protective aprons varied by departments (Table 3).

**Table 3:** Percentage of days with correct use of personal protective equipment (PPE) by porters in Bwindi Community Hospital, Uganda, October 2017.

	**Sexual reproductive health department**	**Out patients department**	**Adult inpatient department**	**HIV/AIDS department**	**Pediatric ward**
**Personal protective equipment to be used**	**%**	**%**	**%**	**%**	**%**
Wearing plastic apron	81	26	87	13	71
Wearing any type of gloves	100	100	100	100	100
Heavy duty gloves worn	10	21	33	28	27
Wearing a surgical face mask	29	13	29	7	26

*Number of days assessed varied between 29 and 31*.

### Disposal

Bwindi Community Hospital has a secure waste storage site that is ventilated and well fenced, preventing entry by domestic animals, pets, pests including marabou storks (*Leptoptilos crumenifer*), and unauthorized humans. The hospital waste was, at the time of the conception of the study, disposed of by incineration, burying, placenta pit, and open burning, according to the different waste types.

At the time of the study, compostable waste was transported to compost pits within the hospital land, and placentae were buried in a dedicated pit. Inspection of the compost pits showed the same lack of segregation of compost and non-compost waste as was seen in all the departments, with paper and plastics being the most common contaminant, although no hazardous waste was seen.

Placentae were usually quickly disposed of correctly after deliveries and did not remain on the ward. The only still birth in the month of study was taken for burial by the family (Table 4).

**Table 4:** Disposal of placentae and still born infants in Sexual and Reproductive Health department of Bwindi Community Hospital, Uganda, October 2017.

**Placenta disposal**	**Number**
N° of placenta produced during study period	101
N° of placenta disposed of by time of daily inspection	81
**STILL BIRTHS**	
N° of still born infants delivered during study period	1
N° of still born infants buried by time of inspection	1

In October 2017, hospital waste was not disposed of on-site, as previously (incineration, and open burning). Instead, such waste was transported from the storage site to a processing plant in Eastern Uganda by an internationally funded health care waste handling company in a dedicated refrigerated vehicle. This new arrangement has been running since July 2016, after the study was conceived.

The collection and off-site transportation of the non-compostable and hazardous waste by the national contractor was irregular. The hospital understood that waste would be collected every 5 days; however, this was not adhered to. During the month of the study, waste was collected four times out of an anticipated six. The intervals between collections varied between three and seven days. Despite the inconsistency in timing of waste collections, on all occasions all stored waste was removed from the hospital.

But not everyone on the hospital staff thought that such transport was appropriate. “*We should dispose of our waste, not send it away,”* said a clinical staff member.

One non-clinical staff member was concerned about the indiscriminate disposal of clinical waste that arises from incorrect segregation. He said, “*You find blood stained gauze mixed with empty intravenous fluid bottles and urine bags*.”

Ionizing radiation in x-ray waste was a concern as identified by a senior clinical officer, who said, “*There seems to be no clear way of handling ionizing waste*.” It proved impossible to quantify the laboratory and X-ray chemical waste, since the fluids were disposed of directly down the drains. These drains run into deep soakaways under grassed areas and were separate from other drainage systems. It was not known how the continued use of such soakaways over years had contaminated the local groundwater, which drains into the river, which in turn is used as a water supply by humans and animals.

### Risk

The respondents correctly identified a number of risks that include cross infection (“*waste that is infectious contains pathogens”* clinical officer*)*, occupational hazards, and direct injury (“*can cause harm to health care workers and patients*” clinical staff), pollution of the environment (“*leads to environmental pollution”* clinical staff; “*minimize contamination of water… reduce air pollution”* non-clinical staff). These risks can affect people immediately or in the future, directly or indirectly.

A non-clinical staff member said that safe handling of hazardous waste is of medium priority because of the limitation of funds availed for such activities, but was quick to note that this topic should be highly prioritized because of the risks involved. This recognizes that there is a degree of risk for all staff who are involved in hazardous waste management.

### Concerns

Surprisingly, a few staff had no concerns about the waste management of their department or the hospital. “*I don't have any concerns,”* commented a clinical staff member, while another said, “*Hazardous waste is handled very well”. One* non-clinical staff member had an understanding of the size of the issue the hospital faces: “[The] *issue is not hazardous waste, but the issue is [all] waste*.”

However, many of the concerns expressed centered on the porters. Their knowledge about waste management, especially waste handling was seen as not adequate, confirming other findings in this study (Table 3). One clinical officer said, “*The porters are not aware of the dangers of poor waste handling*.” Another said, “*Porters need a refresher about waste management*.” A third commented that, “*[I'm] not sure about the immunization status of porters against Hepatitis B*.” The porters were mainly using soft medical disposable gloves, which concerned a clinical officer who emphasized that porters should be given heavy duty gloves.

Some of those who expressed concern about the porters were less aware of their own responsibility to segregate waste properly at source.

## Discussion

### Summary of the Findings

Over five tons of health care waste was produced in the month observed. Only 28% was clinical waste, while the remainder was compostable waste from food preparation by patients or their relatives. Clinical staff had a good awareness about health care waste management. Unfortunately, this did not translate into proper segregation of waste into the different categories at the point of generation. Non-clinical staff involved in health care waste management had limited awareness of the risks involved in their roles. Their incorrect use of personal protective equipment while transporting the waste put them at risk of infection as well as occupationally-induced issues such as back problems. Disposal of chemicals directly into the ground posed a potential risk to water sources.

### Strengths of the Study

Strengths of the study include following the complete waste disposal process within the hospital from waste generation to removal from the site for disposal. We also assessed staff awareness and practice about waste management. Data was collected for a whole month.

A weakness was that only five out of eight hospital departments were assessed and no other health centers or service delivery points were included. Details of quantities and kinds of waste fluids disposed of by pouring down drains and where the soak-away may drain to were not available, so the safety of fluid waste disposal could not be effectively assessed. This needs further work.

### Reasons for Findings

The considerable amount of compostable waste from the Sexual and Reproductive Department (SRD) and adult inpatients was generated by relatives providing meals for the large number of in-patients, including 28 beds reserved for the use of pregnant women living in the hospital while awaiting delivery. The food waste includes bulky plantain skins from preparation of the local staple, bananas (matooke).

The relatively large amount of hazardous (highly infectious + infectious) produced by SRD (including maternity) were due to placentae and blood-contaminated materials from deliveries. The placentae not removed at the time of inspection indicate the on-going nature of deliveries, not the inadequacy of removal (Table 4).

A large percentage of the hazardous waste from the HIV department was from items contaminated by body fluids during patient investigations.

Poor segregation of waste unnecessarily increased the amounts of apparently hazardous waste, and therefore the cost of disposal, whether to the hospital directly, or as at present to the private internationally funded waste company. The issue of incorrect segregation means that waste can be disposed of incorrectly. This is still true now that both non-compostable and hazardous waste are transported from the storage site to the processing plant in Eastern Uganda.

### Comparison of Findings

The proportions of hazardous (12%) and non-hazardous waste (87%) was similar to that reported in other low- and middle-income countries (22, 23). Segregation was incorrect across all departments; this is a common problem reported in other studies (24, 25). From the interviews it was clear that clinical staff did not entirely apply the knowledge they had during segregation of waste in all departmental generation points, as found elsewhere (26–28). This is complicated by the lack of supply of the correctly colored bin liners to BCH.

The poor segregation and handling of waste increased the risk of infection to staff, patients, and visitors (9). Cross infection was taken seriously in both the hospital and the community health centers, with a dedicated infection control committee which is ready to act on conclusions of the study (28).

Overall, in our clinical areas, sharps were well handled, although globally sharps contribute the biggest morbidity of the waste (29).

Transportation within the hospital to the storage area was done manually by porters who did not use personal protective equipment correctly. This practice increases the risk of direct contact with contaminated waste and of injuries from sharps and also of spills of waste from the bin liners to the pathways and the compound. It is of note that the porters' basic knowledge about all aspects of proper waste handling was severely limited (30).

Water source contamination by chemicals from laboratory and X-ray has also been described in Haiti (31). Contamination of water sources may affect livestock and humans directly through drinking, and farming fields through irrigation, which is very important because the local community largely depends on agriculture for its livelihood. In Bwindi Community Hospital, while we do not know the existence or extent of any water contamination, the study has highlighted the need to investigate this in the future.

Human to animal spread of infection has been documented many times [reviewed in Chartier (16)]. Cross contamination leading to transmission of infection in the catchment area of the hospital and community project could lead on to exposure, directly, or indirectly through intermediary species, not only of the human population but of the gorilla (*Gorilla beringei beringei*) population in the adjacent world heritage site (13). Environmental degradation, particularly deforestation (14), enhanced by waste pollution, may further endanger the nearby gorilla population, a responsibility the hospital is taking increasingly seriously.

### Lessons Learnt

Knowledge of clinical staff is largely adequate with regard to the importance of recognizing the different waste types. Unfortunately, except for sharps and vials, this is not applied in the practical management of waste in the hospital.

Non-clinical staff involved in waste handling show little understanding of the resultant risks leading to possible adverse occupational outcomes and hospital contamination. As a result of this study, the hospital management more clearly recognized the risks to the health of the wider community, the natural environment, including contamination of water sources, and even possibly cross infection to local wild animals.

### Implications

There is a need for the hospital to develop systematic methods to improve waste management for the benefit of staff, patients, and the wider community. This could be achieved through three approaches: education, audit, and review of the drain design.

- Continuous education for all hospital staff about safe and proper waste management with emphasis on segregation at point source, PPE, transport, storage, and disposal. Staff need to realize that they are the primary stake holders in ensuring that a clean and safe working environment. Clinical and non-clinical staff should contain a regular component on waste management.
- Waste audits should become more regular and consistent. Collection by the waste handling company for offsite management should be monitored to ensure consistency to avoid prolonged stay of waste which would lead to scavenging by rodents. Periodic close monitoring and evaluation of waste management would impart a sense of security against occupational health risks, increasing the moral among hospital workers.
- The procedures for disposal of potentially hazardous liquid waste draining into the ground should be reviewed. Liquid waste could be treated by dilution and liquid treatment before disposal. The hospital may need some investment to re-engineer the waste flow.

The hospital should prioritize health care waste management with dedicated budget line allocations. Over three tons of compostable waste was produced in 1 month. How this could be better managed to support the local agricultural community requires further work. Subsequent monitoring and auditing of the waste management protocols and policies will improve resources and ensure a cleaner and safer health care institution and the surrounding environment.

## Conclusion

Health care waste management at Bwindi Community Hospital still faces many challenges and does not meet WHO standards that would ensure safety for staffs, clients, and the surrounding environment from hospital-related infections. The five departments in the study produced over 5,000 kg of waste in 1 month, a large amount that needs to be properly managed to minimize infections, water source contamination, and environmental pollution.

The hospital should arrange sufficient on-going training programs for clinical and non-clinical staff, and use of personal protective equipment by porters should be emphasized. Efforts should be made to improve the minimization of waste at source. Audit of waste management across the hospital, as well as re-engineering for the chemical wastes, is needed to ensure the lessons learned in this study are not lost but built into BCH's waste management policy and practice.

## Data Availability

All datasets generated for this study are included in the manuscript and/or the supplementary files.

## Ethics Statement

Ethics approval was obtained from the Bwindi Community Hospital Health and Scientific Committee local Ethics Committee, and also from the Ethics Advisory Group of International Union Against Tuberculosis and Chronic Lung Disease, Paris, France.

## Author Contributions

SK conceived the study. SK, EW, AS, AD, and BM designed the study protocol and all authors read and approved the study protocol. SK collected the data. All authors contributed to analyzing and interpreting the data. SK and AS drafted the manuscript and all authors critically revised the manuscript for intellectual content. All authors read and approved the final manuscript. SK and BM are guarantors of the paper.

### Conflict of Interest Statement

The authors declare that the research was conducted in the absence of any commercial or financial relationships that could be construed as a potential conflict of interest.
